# Indications and complications of lower extremity amputations in two tertiary hospitals in the North West Region of Cameroon

**DOI:** 10.11604/pamj.2023.44.196.34969

**Published:** 2023-04-20

**Authors:** Ntuntu Sweni Tamfu, Tsiaguadigui Jean Gustave, Etienne Ngeh Ngeh, Njobe Brice Kwijirba, Pisoh Tangnyin Christopher

**Affiliations:** 1 Department of Clinical Sciences, Faculty of Health Sciences, University of Bamenda, Bamenda, Cameroon,; 2 Faculty of Medicine and Pharmaceutical Sciences, University of Douala, Douala, Cameroon,; 3 Regional Hospital Bamenda, Bamenda, Cameroon,; 4 Research Organisation for Health Education and Rehabilitation, Yaoundé, Cameroon,; 5 Department of Physiotherapy St. Louis University Douala, Douala, Cameroon,; 6 Université Libre de Bruxelles, Bruxelles, Belgique

**Keywords:** Lower extremity amputation, indications, types of amputation, complications, North West Region, Cameroon

## Abstract

**Introduction:**

the study aimed to outline the common indications and complications of lower extremity amputations among amputated patients in two tertiary hospitals in the North West Region of Cameroon.

**Methods:**

this hospital based retrospective study was conducted in the Bamenda Regional Hospital and Mbingo Baptist Hospital over a 5-year period from 2015-2019. We identified and reviewed amputee´s medical records over the 5-year period. A well designed extraction form was used for data collection and the data obtained was analysed using Epi-info version 7.2.4.

**Results:**

a total of 148 patients underwent 159 amputations in Bamenda Regional Hospital and Mbingo Baptist Hospital with a mean age of 54.28 years (SD ±19.28). Males out-numbered females with a ratio of 2: 1. The most common indication for lower extremity amputation was Diabetic foot gangrene (42.14%) followed by trauma (22.01%). The most frequently performed procedure was Below Knee Amputation (48.42%). Post-amputation complication rate was recorded at 40.25% with surgical site infection being the most common (25.8%). Mortality rate was recorded at 6.28%.

**Conclusion:**

diabetic foot gangrene is the leading indication of lower limb amputation in our setting and the most frequently performed procedure is Below Knee Amputation with surgical site infection being the most common complication.

## Introduction

Amputation, from the Latin word *amputare* meaning to “cut out” is the surgical removal of all or part of a limb by cutting through bone or joint [1,2]. As a surgical procedure, amputation dates back to the days of Hippocrates when it was performed for several reasons including punitive, ritualistic and therapeutic reasons [1-4]. Today lower extremity amputation (LEA) remains an important lifesaving surgical procedure often required when limb savage is impossible or when the limb is dead, dying, viable but non-functional or endangers the patient´s life [5]. Its indications vary between and within countries with most commonly encountered indications being trauma, complications of diabetes mellitus and peripheral vascular disease [6-8]. While some authors found trauma to be the most common indication of LEA, others have reported complications of diabetes mellitus as the leading cause of amputation in Sub-Saharan Africa [9-14]. One of the ways of addressing the observed discrepancies is to carry out more studies to generate enough data on the subject. Furthermore, this lifesaving surgical procedure is often associated with profound economic, social and psychological burden especially in our setting with limited centers with the capacity to provide adequate care rehabilitation [8]. Therefore, the most crucial need is to identify factors that may lead to a decrease in the amputation rate and an improvement in rehabilitation of patients [5]. In addition, there is limited literature on LEA in Cameroon in general and the North-West Region in particular. This study aims to determine the pattern and complications of LEA in two tertiary hospitals in the North West Region of Cameroon.

## Methods

**Study design and setting**: this was a hospital-based retrospective study conducted at Bamenda Regional Hospital located in Mankon in the Bamenda Health District and at Mbingo Baptist Hospital located in Mbingo Village in Kom. Mbingo Baptist Hospital is a reference hospital for orthopaedic pathologies in the region and is known for its outstanding services in orthopaedics whilst Bamenda Regional Hospital is the top government referral hospital in the region. The study was conducted over a six months´ period extending from March to September 2020. We reviewed the medical records of patients amputated over a 5-year period, from January 1^st^, 2015 to December 31^st^, 2019.

**Study population**: the study population consisted of medical files of those with LEA in two tertiary hospitals. We included all accessible medical files of amputees with LEA and excluded those with upper extremity amputations. Other exclusion criteria included LEA files with incomplete data and files of amputees where the amputation was conducted elsewhere referred for followed-up in the two hospitals.

**Sample estimation**: a convenient sampling method was used.

**Data collection and analysis**: a data collection grid was used to extract socio-demographic (age, gender and employment status), indications (diabetic foot gangrene, traumatic injuries, peripheral vascular diseases, infections, tumours and chronic ulcer without definitive diagnosis), types of lower limb amputation (above knee, knee, below knee, ankle, trans-metatarsal and toe disarticulations) and post-amputation complications (surgical site infections, phantom limb pain, wound necrosis, wound dehiscence, re-amputation…etc.) data from the patients´ medical files and theatre records. Data collected was analysed with Epi-info v. 7.2.4 for windows and categorical variables were summarized as frequency and percentage while continuous variables were summarized as means, median and standard deviation (SD) and the results reported as per objective.

**Ethical considerations**: approval was obtained from the Institutional Review Board of the University of Bamenda (Project Identification Number: 2020/0233H/UBa/IRB) and from the Cameroon Baptist Convention Health Board Institution Review Board (Institution Review Board study number: IRB2020-15). Confidentiality was ensured by assigning unique identification code to each medical file, participant´s names were not recorded and the collected data was in keeping of the principal investigator.

## Results

A total of 161 files were registered among which 13 were excluded for incomplete data. Thus, a total number of 148 patients underwent 159 lower extremity amputations in Bamenda Regional Hospital and Mbingo Baptist Hospital between January 1^st^, 2015 to December 31^st^, 2019 among which nine (09) patients had a repeat amputation and 2 had bilateral amputation. There were 107 (67.3%) males and 52 (32.7%) females giving a male-to-female ratio of 2: 1. Majority (54.72%) of patients were self-employed in activities like farming, trading, driving (Table 1). The mean age of patients amputated was 54.28 with a standard deviation of ±19.28. The age group 41 - 60 years registered the highest number of amputations (41.51%) as seen in Table 1 below. The case reviewed showed that diabetic foot gangrene was the most common indication of amputations (42.14%) followed by trauma (22.01%) (Table 2). Among patients amputated for trauma the most common cause of trauma was Road Traffic Accident (RTA) resulting in crush injuries, followed by blast injuries resulting from landmines and heavy explosives (Table 2). Chronic leg ulcers and tumours were the least indications of LEA. In addition, infections accounted for some cases of amputations and the most common causes of infections amputations were necrotising soft tissue infections, chronic osteomyelitis and gas gangrene.

**Table 1:** socio-demographic characteristics of patients amputated in two tertiary hospitals in the North West Region of Cameroon

Variables		
Sex	Frequency	Percentage%
Male	107	67.30
Female		
Employment status	52	32.70
Self-employed	87	54.72
Formally employed	15	9.43
Retired	44	27.67
**Student**		
Age group distribution	13	8.18%
≤ 20	10	6.29
21 - 4	24	5.09
41-60	66	41.51
61-80	50	31.45
> 80	9	5.66

**Table 2:** indications of lower extremity amputations in two tertiary hospitals in the North West Region of Cameroon

Indication	Frequency	Percentage (%)
Chronic non healing ulcer	9	5.66%
Diabetic foot gangrene	67	42.14%
Infection	14	8.81%
Chronic osteomyelitis	3	
NSTI	10	
Gas gangrene	1	
Trauma	35	22.01%
Road Traffic Accident	16	
Domestic injury	4	
Warfare (Blast) injury	8	
Ballistic (Gunshot)injury	6	
Burn	1	
Peripheral Vascular Disease	24	15.72%
Tumour	9	5.66%
**Total**	159	100.00%

NSTI: Necrotising Soft Tissue Infection

**Figure 1:**
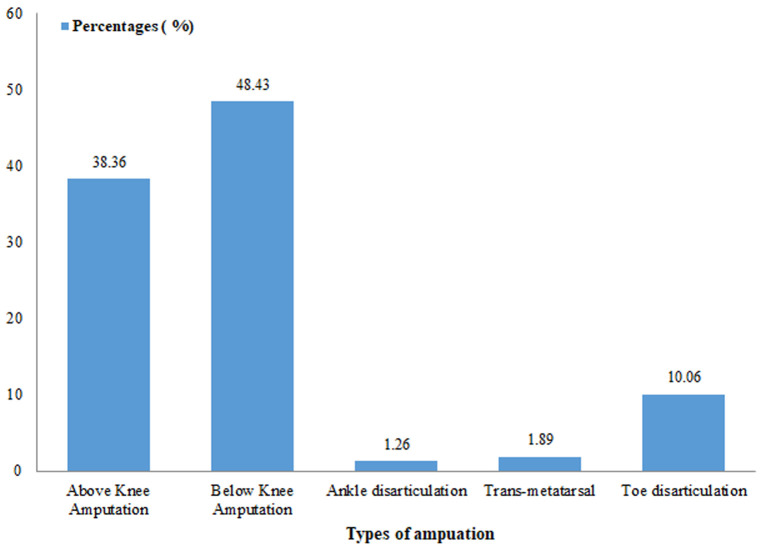
types of lower extremity amputations performed in two tertiary hospitals in the North West Region of Cameroon

**Figure 2:**
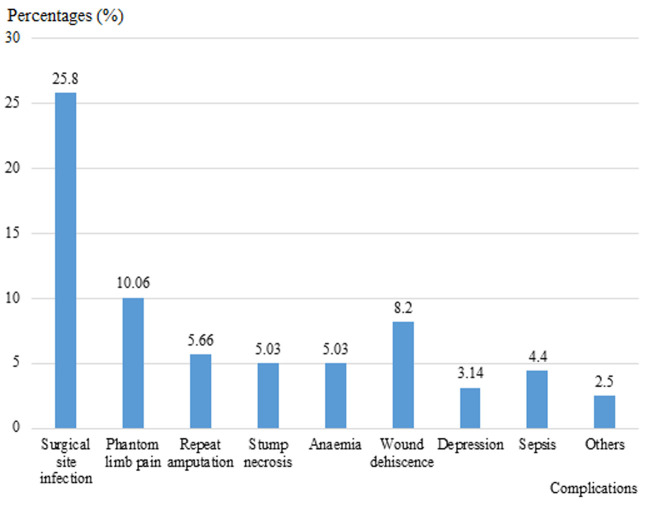
complications of lower extremity amputations in two tertiary hospitals in the North West Region of Cameroon

In this study, the indications of amputations varied among patients of different age groups. Diabetic foot gangrene appears to be the main cause of LEA in people above 40-year-old, whereas trauma followed by malignant tumours is the main reasons of amputation in people below the age of 40 (Table 3). Peripheral Vascular Disease (PVD) is more commonly seen in people above 60 years of age (Table 3). The study revealed that 71.07% of amputee spent weeks to months or years at home before the first presentation to the hospital following the onset of the symptoms and 28.93% spent hours to days. The most common type of amputation registered were major amputation accounting for 138 (86.78%) amputations among which 61 (38.36%) above knee and 77 (48.42%) below knee (trans-tibial) as seen on Figure 1 below. Minor amputations were less performed and represented 13.22% (21 cases) of all lower limb amputations registered and toe disarticulation was the main amputation done in this group with 16 (10.06%) cases followed by trans-metatarsal amputation registering 3 (1.89%) and ankle disarticulation with 2 (1.26%) cases. Post-amputation complication rate was recorded at 40.25% with surgical site infection being the most common complication (25.8%) followed by phantom limb pain (10.06%) (Figure 2).

**Table 3:** distribution of indication of amputation according to the different age groups among patients amputated in two tertiary hospitals in the North West Region of Cameroon

Age group	Diabetic foot gangrene	PVD	Trauma	Infection	Chronic non healing ulcer	Tumor	Total
≤ 20	0	0	**9**	0	0	**1**	10(6.29%)
21-40	0	0	17	1	1	5	24(15.09%)
41-60	42	7	6	4	5	2	66(41.51%)
61-80	20	17	3	7	2	1	50(31.45%)
> 80	5	1	0	2	1	0	9(5.66%)
**Total**	67	24	35	14	9	10	159 (100%)

PVD: Peripheral Vascular Disease

## Discussion

Amputation is a surgical removal of all or part of a limb [9]. It is a lifesaving procedure which is often poses profound economic, social and psychological effects on patient and family [5]. Amputation is therefore a major health problem especially in our setting where there are limited centers with the capacity to provide adequate care and rehabilitation for patients with amputated limbs. As its indications differ between countries and within countries, this study was undertaken to describe the indications, different types of amputations and to determine the post-operative complications of LEA.

**Socio-demographic characteristics**: this was a retrospective study consisting of describing prospectively collected data. During the study period, 159 lower limb amputations were done. The male preponderance in this study is in keeping with the findings of other studies [1,6,9,11-13,15-17]. The male to female ratio of 2: 1 and the age distribution which ranged from 1 to 115 years with a mean age (standard deviation) of 54.28 (±19.28) was comparable to findings reported in 2013 in Rwanda where the male to female ratio was 2.03: 1 and age range was from 1 to 93 years with mean age (standard deviation) of 54.4 (±18) years and to that of other African authors [3,9,13]. In addition, majority of these patients were in the 4^th^, 5^th^, and 6^th^ decades which is comparable with other studies [1,13,16]. However, this age distribution contrast with other studies reporting peak age in the 3^rd^ and 4^th^, decades including Alegbeleye *et al*. in Cameroon [11]. This variation in age distribution can be explained by the difference in pathologies leading to amputation which varies between hospitals.

**Indications**: in this study, diabetic foot gangrene accounted for 42.14% of amputations followed by trauma found in 22.01% of case and the third most common indication was PVD present in 15.72% of amputations. The trend reported in this study is in agreement with that reported by other authors [13,18,19]. These findings are also consistent with similar study conducted in Nnewi-Nigeria in 2001 which reported that 45.6% of amputations were as a results of diabetic foot gangrene followed by trauma accounting for 19.6% of amputation [16]. In the same line, these agree to results documented by Jawaid *et al*. in Pakistan in 2007 who recounted diabetic foot gangrene as the main indication of LEA accounting for 54.7% of LEA [1]. In addition to this, other African and non-African authors including: Al Agha *et al*., Kayssi *et al*., and Maduagwet *et al*. also report diabetic complications as the leading indication of lower limb amputations [10,14,20]. On the other hand, the results of this study differ from that reported in a study done in Kumbo-Cameroon in 2020 which reported trauma as the leading cause of LEA followed by diabetic foot gangrene which was responsible for 22.09% of amputation [11]. In agreement with to this, Akiode *et al*., and also reported trauma as the leading indication of LEA [9,20]. Another study reported diabetic foot as one of the least indication of amputation and other consider PVD as the leading indication of amputation whereas others reports malignant tumours as the most common indication of LEA [3]. The socio-economic status of the amputees registered in our study explains the leading position of diabetic foot gangrene as the most common indication. In this study, 70% were either self-employed in low income earning activities or retired living in the suburbs which concurs with the study conducted in Cameroon in 2010 reporting that patients of low socio-economic group most often live in the rural areas where there are little or no health facilities and these facilities are not likely to have insulin and oral anti-diabetes medications resulting in poor control of diabetes [21]. This report is in accordance with other authors including: Ogeng'o JA *et al*. in Kenya, Akiode O *et al*. and Katchy AU *et al*. in Nigeria [6,12,22]. Furthermore, the difference in age distribution also explains the results differences. Majority amputees registered in this study were above the age of 50 contrary to other studies where most amputee are younger adults. Young adults are usually more agile and adventurous and are therefore at higher risk of injury. This is in agreement with results reported by other authors including: Alegbeleye *et al*., Thanni *et al*., and Nwosuet *et al*. who all studies reported mean age of amputees below 40 [11,17,23].

**Types of amputation**: the most frequently performed procedure in this study was below knee amputation which was done in 48.42% of amputations followed by above knee amputation done in 38.36%. Levels of minor amputation registered included: toe disarticulation done in 10.05% of cases, trans-metatarsal amputation and ankle disarticulation in 1.88% and 1.25% of cases respectively. A similar trend was reported in other series including those reported by the findings of Murwanashyaka who reported a rate of 37.4% in Rwanda and Katchy in Nigeria who also the reported BKA as the most frequently performed procedure with a rate of 39.13% [3,9,12]. These findings are contradicted by other studies which reported BKA as the most frequently performed procedure including the study of Alegbeleye *et al*. with BKA done in 19.2% and AKA seen in 33.7% of cases [6,11]. The results obtain in our study could be explained in part by the duration of symptoms before presentation to the hospital. This in line with other authors who report that late presentation with spreading gangrene or advanced diabetic foot gangrene or malignant lesions that have involved the underlying bones may force the surgeon to opt for a higher level of amputation [24,25].

**Complications of lower extremity amputations**: in our study, 40.25% of amputations had complications among which some amputees had more than one complication. This is similar to a study conducted by Essoh *et al*. in Cote D´Ivoire where he documented a complication rate of 39% [2]. In agreement to other studies, the commonest complication in this study was surgical site infection occurring in 25.8% followed by phantom limb sensation present in 10.06% then wound dehiscence which was observed in 8.2% of amputation [9,26]. In addition to this, Essoh *et al*. also observed a similar trend in their study in Côte d´Ivoire in 2007 where surgical site infections (SSI) was recorded in 26.9% of amputations [2]. Although the same trend, some studies including Shaw *et al*. in their study titled quality of life and complications in lower limb amputees in Tanzania reported higher rates of SSI where SSI recorded 51% of amputations while others authors including Alegbeleye *et al*. reported lower rates of SSI [11,26]. The differences observed in rate of complication could be explained by the severity of complications leading to amputation. Our results are in contrast with other authors including Kooijman *et al*. who reported phantom limb pain as the leading post amputation complication [27]. Repeat amputation was one of the complications registered in this study occurring at a rate of 5.66%. This is lower than that reported by other authors including Kidmas *et al*. in Nigeria (7.4%), Chalya *et al*. in Tanzania (9.9%) and Essoh *et al*. in Cote d´Ivoire (23%) [2,13,25]. This discrepancy could be explained by the complication rate, late presentation to the hospital and the surgeon´s experience. This in agreement with the report of Chalya *et al*. in Tanzania [13]. The mortality rate in the present study is 6.28% which is significantly lower than that reported by others authors including Chalya *et al*. in Tanzania (16.7%), Essoh *et al*. in Cote d´Ivoire (16%) and Kidmas *et al*. Nigeria (12.6%) but higher than that reported by Masood in Pakistan [1,2,13,25]. The reason for this mortality rate in our study was sepsis and pulmonary embolism which was seen in elderly patients.

## Conclusion

Diabetic foot gangrene remains the most common cause for lower limb amputation in our environment follow by trauma. Most of these amputations were preventable with adequate diabetes complication prevention programs. The most frequently performed procedure is below knee amputation and surgical site infection is the most common post amputation complication. **Study limitations**: being a retrospective hospital based study with information obtained from medical record; this study is limited by the quality of data obtained.

### What is known about this topic

- *Lower extremity amputation is a major public problem, especially in developing countries with limited rehabilitation centers;*
- *Indications of lower extremity amputation vary between and within countries*.

### What this study adds

- *Provide data on indications of lower extremity amputation in our sub-region;*
- *Bring to light the different inpatient complications of lower extremity amputations*.
